# The impact of micronutrient status on health: correlation network analysis to understand the role of micronutrients in metabolic-inflammatory processes regulating homeostasis and phenotypic flexibility

**DOI:** 10.1186/s12263-017-0553-7

**Published:** 2017-02-08

**Authors:** Tim J. van den Broek, Bas H. A. Kremer, Marisa Marcondes Rezende, Femke P. M. Hoevenaars, Peter Weber, Ulrich Hoeller, Ben van Ommen, Suzan Wopereis

**Affiliations:** 1 Netherlands Institute for Applied Science (TNO), Research Group Microbiology & Systems Biology, Zeist, The Netherlands; 2 DSM Nutritional Products, Analytical Research Centre and Human Nutrition and Health Department, Basel, Switzerland

**Keywords:** Metabolic challenge test, Vitamins, Carotenoids, Phenotypic flexibility, Inflammation, Systems biology, Lipid, Glucose

## Abstract

**Background:**

Vitamins and carotenoids are key micronutrients facilitating the maintenance of health, as evidenced by the increased risk of disease with low intake. Optimal phenotypic flexibility, i.e., the ability to respond to a physiological challenge, is an essential indicator of health status. Therefore, health can be measured by applying a challenge test and monitoring the response of relevant phenotypic processes. In this study, we assessed the correlation of three fat-soluble vitamins, (i.e., vitamin A or retinol, vitamin D_3_, two homologues of vitamin E) and four carotenoids (i.e., α-carotene, β-carotene, β-cryptoxanthin, and lycopene), with characteristics of metabolic and inflammatory parameters at baseline and in response to a nutritional challenge test (NCT) in a group of 36 overweight and obese male subjects, using proteomics and metabolomics platforms. The phenotypic flexibility concept implies that health can be measured by the ability to adapt to a NCT, which may offer a more sensitive way to assess changes in health status of healthy subjects.

**Results:**

Correlation analyses of results after overnight fasting revealed a rather evenly distributed network in a number of relatively strong correlations per micronutrient, with minor overlap between correlation profiles of each compound. Correlation analyses of challenge response profiles for metabolite and protein parameters with micronutrient status revealed a network that is more skewed towards α-carotene and γ-tocopherol suggesting a more prominent role for these micronutrients in the maintenance of phenotypic flexibility. Comparison of the networks revealed that there is merely overlap of two parameters (inositol and oleic acid (C18:1)) affirming that there is a specific biomarker response profile upon NCT.

**Conclusions:**

Our study shows that applying the challenge test concept is able to reveal previously unidentified correlations between specific micronutrients and health-related processes, with potential relevance for maintenance of health that were not observed by correlating homeostatic measurements. This approach will contribute to insights on the influence of micronutrients on health and help to create efficient micronutrient intervention programs.

**Electronic supplementary material:**

The online version of this article (doi:10.1186/s12263-017-0553-7) contains supplementary material, which is available to authorized users.

## Background

Vitamins are defined as organic substances that cannot or can only be partially synthesized by the body for the maintenance of health and well-being throughout every stage of life from conception to old age [1]. Many micronutrients, including the A and E vitamins, are involved in the homeostatic regulation of physiological processes intimately involved in whole body metabolism, including oxidative-reductive and inflammatory pathways [2]. Carotenoids are a class of natural, fat-soluble pigments found principally in plants. They have potential antioxidant properties because of their chemical structure and interaction with biological membranes [3]. Chronic insufficiencies in micronutrients including vitamins may contribute to risks of poor growth, cognitive development, morbidity, and mortality [4, 5]. Over the past century, our understanding of the role of nutrition, including that of vitamins, in relation to health has made tremendous progress. This was primarily achieved by a combination of epidemiological studies assessing micronutrient intake and in vitro and in vivo experiments focusing on single targets or pathways affected by single micronutrients. As a result, significant improvements have been made in meeting the needs of undernourished populations for vitamins and other micronutrients resulting in increased intake and the associated improvements in health [6].

To elevate the level of understanding in the science of micronutrient biology and thereby the caliber of intervention programs targeting the undernourished populations, the nutrition field faces major challenges. The first is to establish relevant status biomarkers for micronutrient use in epidemiological studies and intervention studies [7]. For many micronutrients, biochemical assessment of plasma concentrations is of limited value due to strict homeostatic regulation of circulating micronutrient levels and/or large body stores that are not reflected by circulating concentrations. Therefore, there is a clear need for reliable biomarkers that provide a true understanding of biochemical processes intimately linked to micronutrients statuses, e.g., transport or enzyme activities.

Secondly, our current knowledge of micronutrient biology is largely based on the classic definition of health as “a state of complete physical, mental and social well-being and not merely the absence of disease and infirmity” [8]. This definition is in line with much of the nutritional research measuring and correlating states in observational studies or shifting states in intervention studies. Recently, experts in the field have adapted the definition of health to the ability to adapt and self-manage in the face of social, physical, and emotional challenges such as infections, metabolic stress, and mental stress [9, 10]. In this regard, it is important to assess health not only by static measurements but also as the ability to respond in a healthy, resilient manner to relevant challenges, or in other words, to measure “phenotypic flexibility” [11, 12]. By default, this means that health effects of interventions should be assessed not only by shifting states but also in modulation of challenge responses towards a more healthy physiological response. In recent years, it has become clear that perturbation of homeostasis by using nutritional challenge tests (NCT) is informative to quantify health, its related processes, and how this is influenced by food and nutrition [13–16]. Optimal phenotypic flexibility is driven by a well-orchestrated physiological machinery allowing the organism to adapt to the continuously changing environment, of which food plays a major role. Processes and mechanisms forming the basis of phenotypic flexibility include substrate fluxes for ATP production and biosynthesis, oxidative stress and inflammatory responses, and immune functions as well as DNA repair, apoptosis, etc. [12]. Inadequate functioning of these processes reduces the resilience to daily challenges, culminating in the onset and/or further development of disease. Micronutrients including those assessed in this study are expected to play a key role in the regulation of phenotypic flexibility by facilitating adequate responses to stressors and maintenance of homeostasis [2, 17].

To address the abovementioned challenges in the field of micronutrients, a comprehensive systems nutrition approach has often been proposed [18–23], but to our knowledge, only two studies applying proteomics and/or metabolomics have been published so far [7, 17]. This systems nutrition study is the first to provide in-depth insights into the complexity of correlations of micronutrient status with molecular processes underpinning health based on both static and NCT response data. Plasma status of eight micronutrients has been measured in a group of 36 healthy but overweight or obese male individuals [14, 24]. The concentrations of carotenoids (α-carotene, β-carotene, β-cryptoxanthin, and lycopene), retinol, α- and γ-tocopherol, and 25-hydroxyvitamin D_3_ have been integrated with a total of 153 plasma metabolites, 7 clinical chemistry measures, 79 plasma proteins, and an additional 1150 proteins quantified via SOMAscan after overnight fasting, and in response to a NCT. The results clearly demonstrated that nutritional challenge response analysis results in a differential association of multiple micronutrients with health-related processes as compared to static analysis based on overnight fasting measures.

## Methods

### Study design, execution, and analysis

The execution and analytical methodologies of the nutritional intervention study, including the postprandial challenge response, have previously been described in detail [14, 24]. In short, 36 healthy overweight and obese men (BMI 25.6–34.7 kg/m^2^) with mildly elevated C-reactive protein (CRP) levels (1.0–8.1 mg/L) with the aim to focus on low-grade chronic inflammation instead of acute inflammation were included in a double-blind, placebo-controlled, crossover study with test treatment periods of 5 weeks to assess the effects of an anti-inflammatory dietary mixture (resveratrol, green tea extract, a-tocopherol, vitamin C, n-3 (omega-3) polyunsaturated fatty acids, and tomato extract provided in hard and soft gel capsules). For the purpose of this paper, we only included the placebo data in subsequent analysis. Placebo hard and soft gel capsules contained 365 mg microcrystalline cellulose (Microz Food Supplements) and 1360 mg soy lecithin (soya lecithin; Solgar Vitamin and Herb), respectively. At the end of each test period, a NCT, i.e., 500 ml dairy shake (fat 58.7 E%), comprising 300 ml custard, 150 ml cream cheese, and 50 ml whipping cream, was ingested after a standardized fasting protocol (Additional file 1: Table S1 [14]). After an overnight fast, the subjects received a light standardized breakfast. After at least a 4-h fast (except water), they were offered the dairy shake. At time points 0 (fasting condition), 30, 60, 120, 180, 240, and 360 min after the dairy shake, plasma samples were collected and were stored at −80 °C until further use. The study was performed according to the International Conference on Harmonization of Technical Requirements for Registration of Pharmaceuticals for Human Use; guidelines for Good Clinical Practice; the Helsinki Declaration of 1975, as revised in 2000; and the Dutch Regulations on Medical Research involving Human Subjects (WMO, 1999 as revised in 2000). The study protocol had been approved by the independent Medical Ethics Committee METOPP located in Tilburg, The Netherlands. The study was conducted between December 2006 and June 2007 and was registered on ClinicalTrials.gov with identifier: NCT00655798. Detailed description of methods and analyses on metabolic profiling GC-MS (153 plasma metabolites), multiplex proteome analysis by Rules-Based Medicine Inc. (79 plasma proteins), and clinical chemistry measurements (7 parameters), have been previously described [14, 24]. In the context of this manuscript, additional in-depth proteomics was performed using the SOMAscan® platform (1150 proteins, SomaLogic Inc.). In addition, vitamin and carotenoid concentrations have been determined as described below.

### Proteomics

In addition to the previously applied Rules-Based Medicine platform, in-depth proteomics analysis using SOMAscan technology was performed on all EDTA plasma samples. Plasma samples (65 μl) were analyzed on the SOMAscan Version 3 assay, which measures 1150 proteins simultaneously using novel modified DNA aptamers called SOMAmer® reagents to specifically bind protein targets in biologic samples [25–27]. Each sample in this study was normalized by aligning the median of each sample to a common reference. Inter-plate calibration was done by applying a multiplicative scaling coefficient to each SOMAmer reagent. These scaling factors were calculated using the eight reference calibrators on each plate.

### Vitamin and carotenoid analysis

For all analyses, EDTA plasma has been used. The levels of 25-hydroxyvitamin D_3_ have been analyzed by HPLC-MS/MS using a modified, previously published stable isotope dilution assay [28]. In brief, proteins were removed from the plasma with the double volume of acetonitrile, which contained deuterated 25-hydroxyvitamin D_3_ as internal standard. After centrifugation, an aliquot of the supernatant was injected into the HPLC system. Detection of specific mass transitions (MRM mode) of the analyte and internal standard using a triple quadrupole detector (LC/MS/MS) with an atmospheric pressure photo ionization (APPI) source was used for quantification. Levels of retinol and tocopherols have been analyzed using HPLC with fluorescence detection, whereas carotenoids were detected in the same chromatographic run by Vis-detection [29]. In brief, plasma proteins were precipitated with ethanol. Carotenoids, retinol, and tocopherols were extracted from the aqueous suspension with -hexane/BHT. After centrifugation, an aliquot of the organic phase was dried, re-dissolved, and injected onto a reversed-phase (C18) HPLC system.

### Data analysis

All previously and newly generated data were integrated and correlated to concentrations for each of the eight individual micronutrients. Correlation analysis was performed using non-parametric Spearman’s rank correlation test in order to prevent from focusing on outliers. For correlations at fasting, we selected to present only those significant correlations (*p* value <0.05) with correlation coefficients >|0.5| in order to focus on moderate to strong correlations that were considered most relevant. We used the correlations with coefficients between |0.4| and |0.5| as supporting data for interpretation of the >|0.5| results. To substantiate the identified correlations at fasting, ANOVA analysis was performed to determine differences between low, medium, and high micronutrient concentration groups. For this, the 25th and 75th percentiles were calculated for each compound. A value below or equal to its 25th percentile was defined as group 1, values between the 25th and 75th percentiles were defined as group 2 and a value higher or equal to the 75th percentile was defined as group 3. This resulted in a group assignment for each individual value (groups 1, 2, and 3) for each micronutrient. A one-way ANOVA was applied on the fused data set with micronutrient group as fixed factor. Since eight micronutrients were involved, eight different group assignments were available. So, a one-way ANOVA was applied eight times. Diagnostics, such as a Levene test to test the equality of variances of the residuals and assessing the normality of residuals (skewness, normality, and kurtosis or flat relative to a normal distribution), were used to check if the ANOVA assumptions were valid. If the ANOVA assumptions were not met, the corresponding variable was LOG transformed and the ANOVA analysis was repeated. The ANOVA assumptions were checked again using diagnostics. The null hypothesis (no effect of micronutrient group) was rejected at the 0.05 level of significance.

The SOMAscan analytes measured in response to the NCT were analyzed using a mixed model on time effects. Data was LOG transformed to meet the ANOVA assumptions. FDR correction was used to correct for multiple testing. For parameters with a significant time effect (*p* < 0.05) in response to the NCT, incremental areas under the curve (AUC) were calculated using the first measurement (t0) as reference. We determined both AUC positive relative to baseline (AUCp) and AUC negative relative to baseline (AUCn). For previously generated data, Bakker et al. [24] reported on statistical analysis of GC-MS and protein profiling data using two-way ANOVA. Here, we selected those parameters with a significant time effect. Per parameter either AUCp or AUCn values were selected for further correlation analysis, based on the largest average.

For correlations in response to NCT, we selected to present only those significant correlations (*p* value <0.05) with correlation coefficients for AUCs > |0.4| in order to focus on strong and moderate correlations that were being considered as being most relevant.

Correlation networks were created visualizing all significant and relevant parameters with coefficients above the abovementioned threshold connected to micronutrient nodes using their respective correlations as edges, using Cytoscape software (http://www.cytoscape.org/) [30].

## Results

To assess the influence of micronutrient status on processes relevant to health, we determined the plasma concentrations of retinol, the carotenoids α-carotene, β-carotene, β-cryptoxanthin, and lycopene, α- and γ-tocopherol, and 25-hydroxyvitamin D_3_ in a study population of 36 overweight and obese males with mildly elevated CRP levels. Demographic characteristics and key laboratory values of subjects have been reported previously [24]. Correlation analyses were performed to identify the links between micronutrient status and a total of 1389 variables based on clinical chemistry (*n* = 7), metabolomics (*n* = 153), 79 plasma proteins, and the newly quantified proteins via SOMAscan (*n* = 1150, of which *n* = 54 overlapped with RBM platform) at baseline and in response to NCT. Finally, we investigated the representation of pathways and processes by the plasma parameters that were correlated with one or more micronutrients.

### Vitamin and carotenoid status

**Fig. 1:**
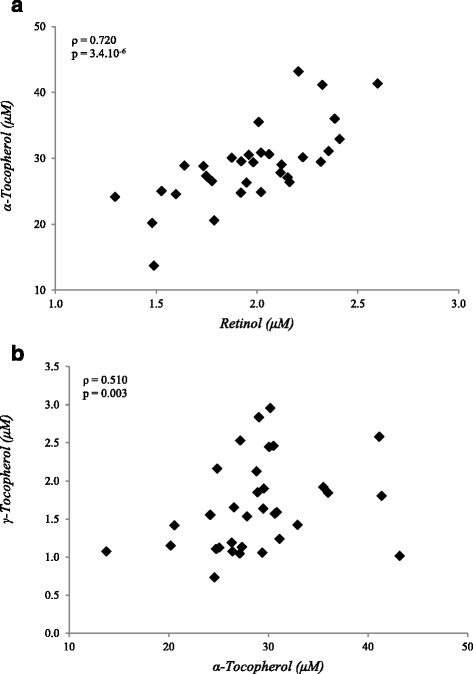
Distribution of plasma concentrations for α-tocopherol vs retinol (**a**) and α-tocopherol vs γ-tocopherol (**b**) in 36 overweight and obese males with mildly elevated CRP levels. Spearman correlation coefficients for both data sets are presented in the figures

Plasma concentrations of the fat-soluble vitamins retinol, α- and γ-tocopherol, and 25-hydroxyvitamin D_3_, and the carotenoids α-carotene, β-carotene, β-cryptoxanthin and lycopene, were assessed at fasting (*t* = 0 min), i.e., before the NCT was applied. The results, as summarized in Table 1, indicate that the study subjects in general have micronutrient statuses within ranges that have been reported in similar studies in Western (male) populations [31–33]. To establish coherence between the eight micronutrients assessed, Spearman correlation analysis was performed on all micronutrient data (Additional file 2: Table S2). This revealed relatively strong correlation between α-tocopherol and retinol status and to a lesser extent between α- and γ-tocopherol status (Fig. 1a, b). Plasma concentrations of the other fat-soluble vitamins and carotenoids did not correlate strongly.

**Table 1:** Plasma vitamin and carotenoid concentrations in 36 overweight men with mildly elevated plasma CRP levels

Vitamin or carotenoid	Plasma concentration (Mean ± SEM)	Range	Number < LOQ (<LOD)	Reference ranges
Retinol (μM)	1.97 ± 0.05	1.29–2.60	Na	1.19–2.24^c^ [31]
γ-Tocopherol (μM)	1.74 ± 0.11	0.73–3.94	Na	0.96–2.30^c^ [31]
α-Tocopherol (μM)	29.0 ± 1.1	13.7–48.3	Na	12–46 [32]
β-Cryptoxanthin (μM)	0.22 ± 0.03	0.06–0.83	Na	0.14–0.40^c^ [31]
Lycopene (μM)	0.62 ± 0.05	0.15–1.28	Na	0.54–0.73^c^ [31]
α-Carotene (μM)	0.06 ± 0.01	<LOD–0.27	16/35 (2/35)^a^	0.07–0.12^c^ [31]
β-Carotene (μM)	0.40 ± 0.03	0.12–0.83	Na	0.31–0.49^c^ [31]
25-Hydroxy vitamin D_3_ (nM)	63.2 ± 5.6	<LOQ–128.8	4/34^b^	20–136.2^d^ [33]

^a^LOQ and LOD for α-carotene are 0.06 and 0.03 μM, respectively

^b^LOQ for 25-hydroxy vitamin D_3_ is 12.0 nM

^c^Mean values from cohorts of healthy adults from five European countries

^d^Mean population values

### Micronutrient status correlation network at fasting

To identify molecular processes and pathways that may be directly or indirectly influenced by status of the eight micronutrients selected, we performed correlation analyses on the 1389 variables quantified at fasting in this study.

**Fig. 2:**
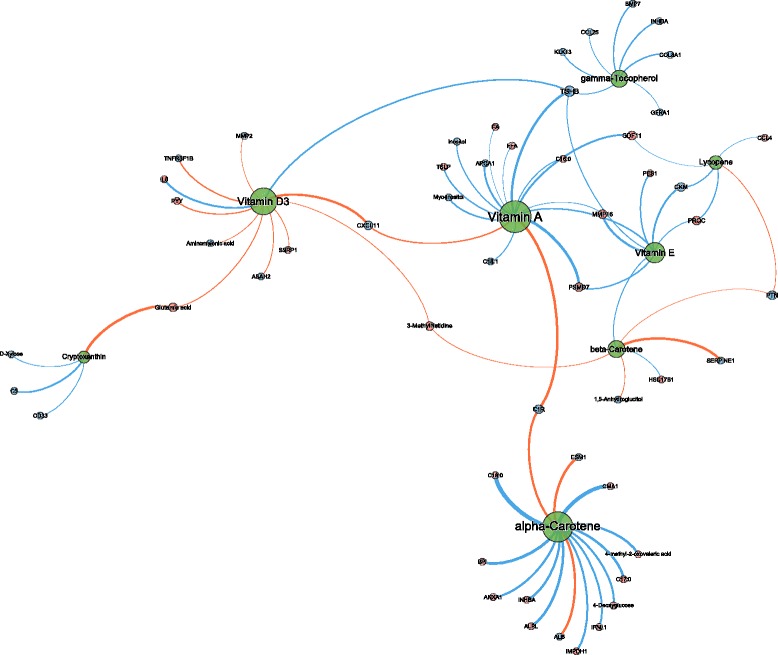
Micronutrient correlation network visualizations of baseline in 36 overweight and obese men with mildly elevated CRP levels. Spearman correlation analysis was performed with all data measured after overnight fasting. The network represent all correlations >|0.5| in connection to the micronutrient nodes. The thickness of edges represents the strength of correlation, positive and negative correlations are presented as *blue and red edges*, respectively. Node size reflects the degree of centrality in the network. Centrality is a measure of the connectedness of a network node; it therefore reflects how many connects each node has to others. Activin A receptor type 1B (ACVR1B), aldo-keto reductase family 1, member A1 (AKR1A1), alanine aminotransferase (ALT), apolipoprotein C3 (APOC3), apolipoprotein H (APOH), ATP synthase H+ transporting mitochondrial F1 complex beta polypeptide (ATP5B), bone morphogenetic protein 1 (BMP1), stearic acid (C18:0), vaccenic acid (C18:1), complement C1r subcomponent (C1R), complement component (C3), CD40 molecule (CD40), checkpoint kinase 1 (CHEK1), chemokine cxc motif ligand 8 (CXCL8), diglyceride (DG), family with sequence similarity 107 member 8 (DRR1), ephrin A5 (EFNA5), estrogen receptor 1 (ESR1), Fc receptor like 3 (FCRL3), homeostatic model assessment for insulin resistance (HOMA-IR), high sensitivity c-reactive protein (hsCRP), interleukin 1 receptor type 1 (IL1R1), interleukin 20 receptor subunit alpha (IL20RA), interleukin 34 (IL34), interleukin 5 (IL5), kallikrein related peptidase 8 (KLK8), kallikrein related peptidase 11 (KLK11), keratin 18 (KRT18), layilin (LAYN), leptin receptor (LEPR), leukocyte immunoglobulin like receptor B2 (LILRB2), leukotriene a4 hydrolase (LTA4H), mitogen activated protein kinase 11 (MAPK11), colony-stimulating factor 1 (macrophage) (M-CSF), matrix metallopeptidase 8 (MMP8), osteomodulin (OMD) plasminogen activator inhibitor 1 (PAI1), protein C (PROC), prostaglandin endoperoxide synthase 2 (PTGS2), peptide YY (PYY), serpin family A member 6 (SERPINA6), SH2 domain containing 1A (SH2D1A), secretory leukocyte peptidase inhibitor (SLPI), sortilin-related vps10 domain containing receptor 2 (SORCS2), tenascin C (TNC), tumor necrosis factor receptor superfamily member 10d (TNFRSF10D), tumor necrosis factor receptor superfamily member 19 (TNFRSF19), topoisomerase (DNA) 1 (TOP1), vascular endothelial growth factor A (VEGFA), X-ray repair complementing defective repair in Chinese hamster cell 6 (XRCC6)

The Spearman correlation coefficients were found to range between −0.681 and 0.685. The distribution of *r*’s was similar for all micronutrients. Only limited numbers of correlation coefficients larger than |0.5| were observed, ranging from 3 (out of 1389 variables) for both carotenes to 17 for α-tocopherol. The correlation network based on all individual variables with coefficients larger than |0.5| with any of the micronutrients is visualized (Fig. 2). For some of the strongest micronutrient correlations, confirmation of the relevance of the observed correlations was obtained by ANOVA analyses for differences between the lowest and highest quartile in micronutrient status, illustrating the validity of the observed correlations (Additional file 3: Figure S1). In-depth analyses of the correlations per individual micronutrient showed that only a small proportion of the variables overlap between the micronutrients (Fig. 2) but revealed a considerable group of micronutrient specific parameters. The correlations per micronutrient group, i.e., the carotenoids, the tocopherols, and vitamin D, are described in more detail below, where parameters with significant (*p* < 0.05) correlation coefficients between |0.4| and |0.5| used as supporting data for biological interpretation are shown in italic.

#### Retinol correlations at fasting

Retinol (vitamin A) concentrations correlated positively with a number of parameters from glucose and lipid metabolism that are associated with metabolic disease, including 4-deoxyglucose, plasma total free fatty acid levels, total ketone bodies, and *total cholesterol levels*. As expected, the products of several retinoic acid inducible genes, i.e., matrix metalloproteinase 8 (MMP8) and soluble leptin receptor (sLep-R) also correlated positively with plasma retinol status. Inverse correlations were observed for proteins reflecting inflammatory processes, including complement subunits (C1r [Additional file 3: Figure S1A], *C4b*, *C5b*), C-reactive protein (CRP, Additional file 3: Figure S1B), and leukocyte immunoglobulin-like receptor B2 (LILRB2), a marker of tolerogenic dendritic cells, which are suggestive of an immunosuppressive effect of vitamin A. The correlation network of retinol showed large similarities with processes that are associated with status of the antioxidant vitamin E (α-tocopherol).

#### Carotenoid correlations at fasting

The status of the 4 different carotenoids quantified, i.e., α- and β-carotene, β-cryptoxanthine, and lycopene, showed largely differing correlation patterns at fasting and only overlapped with retinol to a limited extent. Plasma β-carotene concentrations correlated positively with a number of cholesterol-related lipids, including *LDL cholesterol*, *lysophospatidylcholines* (*LPC*), and *sphingomyelins* (*SM*). In addition, sortilin-related VPS10 domain containing receptor 2 (SORC2), a recently identified neuropeptide receptor, *G protein-coupled receptor associated sorting protein 2* (*GASP2*), and *interleukin 34* (*IL*-*34*) were among the strongest correlating proteins. Inverse correlations were found for both systolic and diastolic blood pressure levels (Additional file 3: Figure S1G), which were highly β-carotene specific. In addition, endothelial function markers plasminogen activator inhibitor-1 (PAI-1, Additional file 3: Figure S1H), *von Willebrand Factor* (*vWF*), and *fibronectin* all correlated negatively with β-carotene concentrations. Together, this suggests that β-carotene concentrations modulate vascular health.

Plasma α-carotene concentrations correlated inversely with a number of interleukin-1 (IL-1) associated proteins, including *IL-1b*, *IL-1R4*, and *IL-1R-accessory protein*, the NF-kB activating protein TRAIL-R4, and *livin*, as well as blood *lymphocyte counts*. Together with several positively correlated factors including IL-34 (Additional file 3: Figure S1I), *chemokine CCL1* (*I-309*), *IL-1sRI*, and *neutrophil counts*, which are all involved in inflammatory action, the results suggest an immuno-modulatory role or effect for α-carotene.

β-Cryptoxanthin plasma concentrations correlated with a couple of key enzymes in the arachidonic acid metabolic pathway, i.e., leukotriene A4 hydrolase (LTHA4; inverse correlation), and *prostaglandin H2 synthase 2* (*PTGS2*, *alias COX-2*; positive correlation), suggesting a modulatory role in inflammatory eicosanoid production, which is further supported by the inverse correlation with C-X-C motif chemokine ligand 8 (CXCL8), and the positive correlation with activated protein C (PROC). In addition, this carotenoid correlated inversely with liver function markers alanine aminotransferase (ALT, Additional file 3: Figure S1N) and *γ-glutamyl transpeptidase* (*GGT*) and liver fibrosis associated marker Tenascin C (TENC4 and TNC).

Similar to β-cryptoxanthin, lycopene concentrations correlated positively with PTGS-2 (Additional file 3: Figure S1K) and *activated protein C* (*PROC*). Lycopene levels also correlated positively with a couple of proteins involved in DNA replication and repair, such as DNA repair protein XRCC6 (alias Ku70), checkpoint kinase 1 (CHK1), and topoisomerase 1 (TOP1, Additional file 3: Figure S1L). Interestingly, the concentrations of a large but specific group of *triglycerides* (*TG*) were inversely correlated to lycopene concentrations. These lipids comprised exclusively of TG species with a carbon number between C40 and C52 and lacking any of the TG species with higher carbon numbers.

#### Vitamin E (α-tocopherol only) correlations at fasting

As indicated above, the correlation network of α-tocopherol showed large similarities with that of retinol. Many lipid moieties, including total cholesterol (Additional file 3: Figure S1C), lysophosphatidylcholines (LPC), diglycerides (DG), and phosphatidylcholines (PC), and associated proteins, e.g., apolipoprotein-CIII (ApoCIII, Additional file 3: Figure S1D), were found to correlate positively with baseline α-tocopherol levels. Also, concentrations of *CRP*, *C1r*, *LILRB2*, and a number of other inflammation associated molecules correlated inversely with α-tocopherol. Unlike retinol, moderate negative correlations with *fasting insulin* levels and the derived *homeostatic model assessment for insulin resistance* (*HOMA-IR*) index were observed in the α-tocopherol network. Interestingly, these glucose metabolism markers correlated stronger with the γ-tocopherol plasma concentrations (e.g., HOMA-IR and insulin, Additional file 3: Figure S1E). Accordingly, negative correlations with *insulin growth factor 1* (*IGF-1*) and *insulin growth factor binding protein 3* (*IGFBP3*) as well as with glucose-associated carbohydrates *ribose* and *maltose* were observed.

#### γ-Tocopherol correlations at fasting

γ-Tocopherol is the main form of vitamin E derived from plant seeds. Compared to α-tocopherol, it exerts different properties that may be important for human health [34]. Triglycerides with medium to high carbon number (C54-C56, Additional file 3: Figure S1F) correlated positively with γ-tocopherol levels. In addition, peptide YY (PYY), matrix metallopeptidase 3 (MMP3), layilin, activin A receptor type 1B (ACVR1B), and interleukin 1 receptor type 1 (IL1R1) showed relatively high correlations with plasma levels of this vitamin, which may all be linked to insulin sensitivity and associated processes.

#### Vitamin D_3_ correlations at fasting

The parameters that correlated positively with fasting 25-hydroxyvitamin D_3_ plasma levels showed considerable overlap with parameters that had a positive correlation to γ-tocopherol levels. The strongest correlations were observed for kallikreins 8 and 11 (Additional file 3: Figure S1O), IL-20 receptor antagonist (IL-20Ra), secretory leukocyte protease inhibitor (SLPI), and ephrin-A5. In addition, vitamin D levels correlated with multiple sphingomyelins (SPMs), indicative of cell turnover and apoptosis. The parameters showing inverse correlations were in general specific to vitamin D, with *IL-3* and 5 (Additional file 3: Figure S1P), *free fatty acid C20:3* (*C20:3-FFA*), estrogen receptor (ER), SH2 domain protein 1A (SH21A) and aldo-keto reductase family 1, member A1 (AK1A1) being the top-ranked proteins. Together, this data suggested a link of vitamin D_3_ levels with inflammatory response and cell viability or apoptosis.

### Micronutrient status correlation network and phenotypic flexibility

**Fig. 3:**
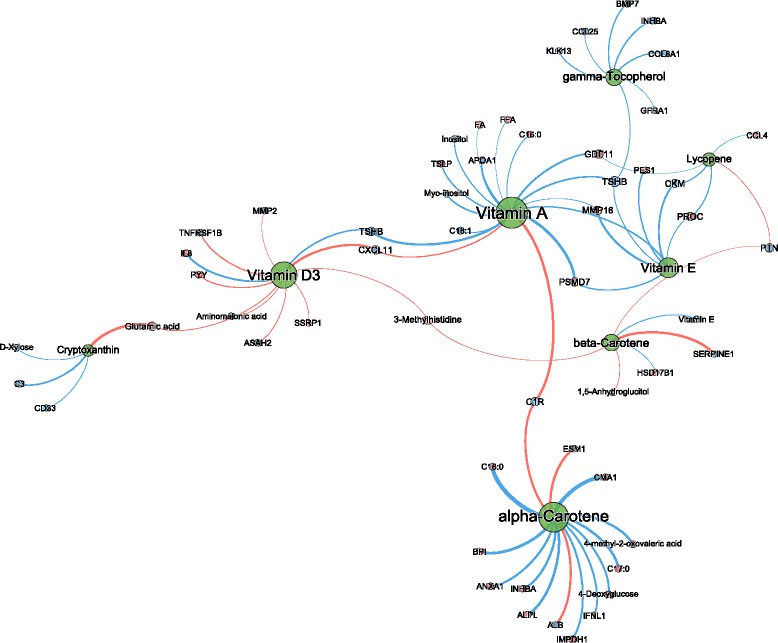
Micronutrient correlation network visualizations of NCT response in 36 overweight and obese men with mildly elevated CRP levels. Spearman correlation analysis was performed with all data measured with micronutrient AUCp or AUCn response data, i.e., NCT response. The networks represent all correlations >|0.4| in connection to the micronutrient nodes (AUCp = blue node, AUCn = red node). The thickness of edges represents the strength of correlation, positive and negative correlations are presented as *blue and red edges*, respectively. Node size reflects the degree of centrality in the network. Centrality is a measure of the connectedness of a network node, it therefore reflects how many connects each node has to others. Albumin (ALB), alkaline phosphatase, liver/bone/kidney (ALPL), annexin A1 (ANXA1), apolipoprotein A1 (APOA1), N-acylsphingosine amidohydrolase (non-lysosomal ceramidase) 2 (ASAH2), bone morphogenetic protein 7 (BMP7), bactericidal/permeability-increasing protein (BPI), palmitic acid (C16:0), margaric acid (C17:0), stearic acid (C18:0), vaccinic acid (C18:1), complement C1r subcomponent (C1R), complement component 3 (C3), C-C motif chemokine ligand 4 (CCL4), C-C motif chemokine ligand 25 (CCL25), CD33 molecule (CD33), creatine kinase m-type (CKM), chymase 1 (CMA1), collagen type VIII alpha 1 (COL8A1), chemokine cxc motif ligand 11 (CXCL11), endothelial cell specific molecule 1 (ESM1), growth differentiation factor 11 (GFD11), GDNF family receptor alpha (GFRA1), hydroxysteroid (17-beta) dehydrogenase 1 (HSD17B1), interferon lambda 1 (IFNL1), interleukin 8 (IL8), Inosine 5′ monophosphate dehydrogenase 1 (IMPDH1), inhibin beta A (INHBA), kallikrein related peptidase 13 (KLK13), matrix metallo peptidase 2 (MMP2) matrix metallo peptidase 16 (MMP16), pescadillo ribosomal biogenesis factor 1 (PES1), protein C (PROC), proteasome 26S subunit, non-ATPase 7 (PSMD7), pleiotrophin (PTN), peptide YY (PYY), serpin family E member 1 (SERPINE1), structure specific recognition protein 1 (SSSRP1), tumor necrosis factor receptor superfamily member 1b (TNFRSF1B), thyroid stimulating hormone beta (TSHB), thymic stromal lymphopoietin (TSLP)

In order to assess the impact of the status of specific vitamins and carotenoids on phenotypic flexibility as a measure of health, we investigated the correlations of plasma status with the response profiles during a NCT. AUCs (either positive or negative) for all parameters that were assessed during the postprandial response with a significant time effect (418 out of 1389 parameters) were correlated with fasting plasma concentrations of each of the eight micronutrients analyzed. Similar to the resulting correlations at fasting, only limited numbers of significant correlations with coefficients larger than |0.4| were observed, showing correlation coefficients ranging between −0.69 and 0.56. A total of 69 correlations fulfilled the criteria, of which 14 with α-carotene, 6 with β-carotene, 4 with cryptoxanthin, 7 with γ-tocopherol, 5 with lycopene, 16 with vitamin A, 11 with vitamin D_3_, and 6 with vitamin E, as visualized in Fig. 3. To our knowledge, many of the parameters in this network have not been associated with any of these micronutrients before, possibly explained by the lack of similar studies investigating the link between vitamins, carotenoids, and nutritional challenge test responses. This is also shown in the fact that there were only nine overlapping correlations with specific metabolites or proteins from the micronutrient response network at fasting. Thus, the micronutrients each generate a nearly complete and unique postprandial response.

Most correlations were found for vitamin A. Lipid metabolism associated parameters such as non-esterified fatty acids (FFA), total free fatty acids (FA), and the fatty acid C16:0 (C16:0), which all showed a positive response to the NCT (AUC+), were inversely correlated to vitamin A status. This indicates that a higher retinol status is associated with a lower continuous increase in plasma FFA concentrations after an initial lag phase of around 2 h to the NCT. Furthermore, free fatty acid C18:1 (C18:1), inositol and myo-inositol known to be involved in phospholipid metabolism and apolipoprotein-A1 (ApoA1) associated with levels of HDL cholesterol, all showed a negative response to the NCT (AUCs were inversely correlated to retinol status). So, a higher vitamin A status is associated with a decreased negative response to the NCT of these lipid metabolism associated parameters. Positive correlations of vitamin A and also α-carotene and vitamin D_3_ status to chemokine cxc motif ligand 11 (CXCL11) and complement C1r subcomponent (C1R) both with AUC, suggest a larger decrease of these inflammatory parameters in response to the nutrient bolus in the case of higher micronutrient concentrations.

α-Carotene shows in general inverse correlations to the measured metabolites and/or proteins as correlation is negative in 11 out of 14 parameters. The response of the most of the parameters is positive upon NCT, as 11 out of 14 parameters showed an AUC increase (red dot, AUC+). This means in general that higher levels of α-carotene are associated with a reduced amplitude of these parameters in response to the NCT.

Saturated free fatty acids C17:0 and C18:0 are increased in response to the NCT as well as 4-methyl-2-oxovaleric acid (promotes insulin secretion from β-cells), 4-deoxyglucose, and the enzymes alkaline phosphatase (ALPL), inosine 5′-monophosphate dehydrogenase 1 (IMPDH1) all showed this inverse correlation, indicating that higher levels of α-carotene are associated with a reduced amplitude of these parameters in response to the NCT. Furthermore, protein levels of chymase 1 (CMA1) and the anti-inflammatory proteins interferon lambda 1 (IFNL1), annexin A1 (ANXA1), and bactericidal permeability-increasing protein (BPI), which is a lipopolysaccharide binding protein, further suggest an immuno-modulatory role for α-carotene. Finally, albumin (ALB) and endothelial cell specific molecule 1 (ESM1) showed a negative response to the NCT, which both showed a positive correlation to α-carotene, meaning that higher levels of α-carotene are associated with a larger reduction of these proteins in response to the NCT.

In contrast, vitamin D_3_ is the only micronutrient which showed almost complete positive correlations with all its associated parameters except for interleukin-8 (IL8) and thyroid-stimulating hormone beta (TSHB), suggesting that vitamin D_3_ is associated with enhanced responses (both negative as well as positive) to the NCT. Reduced vitamin D_3_ serum levels are known to be associated with low-grade inflammatory health states such as obesity. Here, we observed an inverse correlation of vitamin D_3_ with IL8 suggesting that subjects with higher levels of vitamin D_3_ status have a reduced pro-inflammatory response upon NCT. Vitamin D_3_ levels are also associated with increased response of gut hormone peptide YY (PYY), which is indicative for a higher appetite reduction upon the NCT. Furthermore, factors that play a role in impairment of adipogenesis in T2DM such as matrix metallopeptidase 2 (MMP2), tumor necrosis factor receptor superfamily member 1B (TNFRSF1B), and chemokine cxc motif ligand 11 (CXCL11), which all showed reduced levels in response to NCT, showed an enhanced reduction in persons with higher vitamin D_3_ levels.

Micronutrient status of vitamin D_3_ linked to β-carotene via 3-methylhistidine, a marker for muscle protein catabolism, showed increased levels in response to NCT and was observed to have a positive association to both micronutrients. Additionally, β-carotene is also positively associated with enhanced reduced response of metabolite 1,5-anhydroglucitol and of proteins SERPINE1 (PAI-1) and pleiotrophin (PTN) which are all linked to obesity and T2DM.

On the other side of the network, vitamin D_3_ is linked to cryptoxanthin. Both micronutrients have a similar positive correlation with AUCn of glutamic acid in response to NCT. Markers CD3 and complement C3 showed reduced concentrations in response to NCT in combination with a negative correlation, indicating that higher levels of cryptoxanthin are associated with a decrease of these immune modulating markers in response to NCT.

Vitamin E (α-tocopherol) and also γ-tocopherol are both inversely correlated to the response of all associated metabolites and proteins, indicating that levels of both tocopherols reduce the NCT response. Upon administration of the NCT, four of the α-tocopherol associated proteins showed increased concentrations, which were pescadillo ribosomal biogenesis factor 1 (PES1) and proteasome 26S subunit, non-ATPase, 7 (PSMD7), protein c (PROC), and matrix metallopeptidase 16 (MMP16), which all have a proteolytic function.

γ-tocopherol is linked to α-tocopherol and also to vitamin A and vitamin D_3_ via an inverse association to thyroid-stimulating hormone beta (TSHB). Furthermore, levels of γ-tocopherol are associated with a reduced negative response of the proteins bone morphogenetic protein 7 (BMP7), inhibin beta A (INHBA), collagen type VIII alpha 1 (COL8A1), and GDNF family receptor alpha 1 (GFRA1) which are all related to tumor growth factor (TGFβ) that play a role in glucose tolerance regulation. Similar associations have also been observed for kallikrein-related peptidase 13 (KLK13) and C-C motif chemokine ligand 25 (CCL25).

Lycopene shares 2 associations with vitamin E (PROC and CKM) and 1 association with vitamin A (GDF11). The positive response of macrophage inflammatory protein (CCL4) upon NCT is exclusively correlated to lycopene levels. The inverse relation to the micronutrient suggests anti-inflammatory properties for lycopene.

## Discussion

In view of the newly proposed definition of health [10], emphasizing the individual’s ability to adapt to daily life stressors such as excess nutrition, it is of importance to understand the impact of various components influencing physiology in homeostasis and other relevant challenges. Many studies, including those in nutrigenomics, have been performed with a large variety of nutritional challenges to establish the challenge response characteristics in subjects with differential health states as described extensively in a recent review by Dijk-Stroeve [16]. However, this study is the first to assess phenotypic flexibility as measured by nutritional challenge responses in the context of plasma micronutrient status, as well as the first to describe the micronutrient interaction network in homeostasis. We investigated the micronutrient correlation networks in a population of overweight and obese men with mildly elevated plasma CRP levels, indicative of low-grade chronic inflammation, to establish that multiple micronutrients are related to multiple health-related processes that maintain homeostasis and phenotypic flexibility.

Our study population was relatively healthy with respect to their micronutrient statuses; the plasma concentrations of all micronutrients, except for vitamin D, were within proposed ranges of sufficiency based on defined cut-offs or previously reported ranges in the general population [31–33]. We did not find any subjects to be overtly deficient for vitamin E, based on the cut-off value for deficiency in healthy adults (12.0 μM) as proposed by the Institute of Medicine [35] or due to a low ratio of serum α-tocopherol to lipids (<0.8 mg/g total lipids), as well as for retinol, based on a deficiency cut-off value of 0.7 μM. Although the average 25-hydroxyvitamin D status (63.22 ± 5.55 nM) indicates that this study population is sufficient, over 30% of the subjects were below 50 nM, suggesting insufficiency to deficiency for this vitamin [36]. This is in line with a systematic review, which reported 37.3% of the general population to be insufficient and 6.7% to be deficient for vitamin D [33]. For the carotenoids and γ-tocopherol, the levels observed reflected those previously reported in general population [31, 32]. Even though the study population was in general sufficient for most micronutrients, the variation in their concentrations was assumed to be sufficient to provide relevant outputs of correlation analyses. The results showed a strong correlation between retinol and α-tocopherol status in the study population, which may be explained by functional interaction. The oxidized α-tocopheroxyl radicals produced are recycled back to the active reduced form through reduction by other antioxidants, such as retinol [37], although its significance in vivo has been debated [38]. Alternatively, the correlation may simply be explained by dietary co-occurrence or joint transport lipid particles. Our analyses returned only a relatively small number of correlations of relevance. Although this may suggest limited interaction, this could actually reflect the relatively normal plasma micronutrient levels in the study subjects. As it may be expected that the impact of micronutrients on various processes and challenge response is small within these normal ranges, many correlations could be masked by the small study size. At the same time, this also emphasizes the strength of the correlations that were observed within this small data set.

In-depth analysis of the correlation network at fasting revealed several correlations that have not been addressed in the past. In a proteomics study of micronutrient status in undernourished Nepalese children, Cole et al. [7] described proteins that correlated well with retinol, α-tocopherol, and vitamin D_3_. In that population, retinol status correlated with retinol-binding protein 4 (RBP4) and complement C1r, a protease involved in initiating the classical complement cascade [39]. Remarkably, whereas the negative correlation with the latter (Additional file 3: Figure S1A) was also observed in our study, no correlation of RBP4 (*r* = 0.034) with retinol status was observed. This may be explained by the relative adequate retinol levels of our study population as compared to the undernourished Nepalese children (2.00 ± 0.3 μM vs 1.04 ± 0.27 μM) not representing a concentration range in which RBP4 is strongly correlated. The negative correlation between retinol status and CRP levels (Additional file 3: Figure S1B) has also been reported before [40, 41]. Together with the other proteins, these markers support the well-established reported immunosuppressive/anti-inflammatory and immuno-modulatory effects of retinol [42]. The relatively strong correlation of specifically β-carotene with blood pressure (Additional file 3: Figure S1G) has been well established. Higher β-carotene plasma concentrations have been associated with lower systolic and diastolic blood pressure levels in several cross-sectional studies on cardiovascular risk [43–45]. Although the mechanisms are still unclear, the antioxidant function of this carotenoid may impact endothelial health and as such, vascular flexibility. Vitamin E, specifically α-tocopherol, has no specific plasma carrier protein but is primarily associated with low to intermediate density lipoproteins for transport [46]. As expected, positive correlations were found between α-tocopherol status and total cholesterol levels (Additional file 3: Figure S1C), which is the principle component of VLDL [47] and ApoCIII (Additional file 3: Figure S1D), which is the principle component of VLDL [47], one of the first apolipoproteins to be released with vitamin E from the liver [48] was also identified in our study. Other potential biomarkers derived from the Nepalese children cohort, i.e., RGS8 as biomarker for α-tocopherol, and vitamin D binding protein (VDBP) together with Plexin-D1 for vitamin D, were not included in our targeted proteomics platforms. The correlation network of α-tocopherol and retinol showed similarity both in the lipid moieties and in the inflammatory parameters. Although we cannot rule out a partially similar role in processes driving these parameters, the overlap may be a representation of the correlation in plasma levels observed, which has been reported previously [49]. This may be the result of a potentially linked joined transport in association with lipid particles. Finally, the positive correlation we observed between lycopene status and DNA repair protein subunit Ku70 has been previously reported in a study describing the protective effect of lycopene on oxidative stress-induced cell death pancreatic acinar cells [50]. This may be part of the antioxidant function of this carotenoid related to ameliorating oxidative stress-induced DNA damage. Taken together, these findings provided confidence in our findings to confirm the expected associations.

Whereas the correlation network at fasting was rather evenly distributed in a number of relatively strong correlations per micronutrient, the network for the NCT responses was rather skewed towards γ-tocopherol and especially α-carotene besides the similar vitamin A response (Fig. 3), suggesting a more prominent role for these micronutrients in the maintenance of the phenotypic flexibility machinery. Only two parameters (inositol and C18:1) which were correlated to vitamin A showed an identical correlation measured at fasting and in response to NCT. In both situations, vitamin A plays a significant role as it has the most (NCT) or second most (at fasting) correlations. Cryptoxanthin and vitamin E showed a large number of correlations in the fasting state, while the number of correlations at least halved in response to the NCT. This is in contrast to α-carotene that only showed three correlations at fasting conditions while this almost quintupled in response to the NTC. This study shows that applying the challenge test concept is able to reveal previously unidentified correlations between specific micronutrients and health-related processes, with potential relevance for health maintenance indicators that were not observed by correlating measurements at fasting.

The carotenoid lycopene has received much attention in view of its reported beneficial effects on aging and cardiovascular disease development risk [3]. Lycopene is reported to eliminate reactive oxygen species (ROS), inhibits lipid peroxidation, and reinforces the immune system. In our study, we observed in homeostatic conditions that lycopene plasma concentrations mainly overlap with other micronutrients. However, its only unique inverse correlation with C-C motif chemokine ligand 4 (CCL4) relates to the protective effect of lycopene on oxidative stress-induced cellular damage and implicates a potential role in chemoprotection in cancer or other diseases [50, 51]. Recently, higher serum concentrations of lycopene have been associated with higher survival time among participants with metabolic syndrome [52].

In contrast to lycopene, γ-tocopherol has received only limited attention in relation to health benefits. Most of the vitamin E research has focused on α-tocopherol as it has been stated that this is the only considered form of active vitamin E by EFSA [53] and due to its presumed higher biological antioxidant activity [54], even though several studies have indicated that only γ-tocopherol and not α-tocopherol plasma levels were biomarkers for cancer and cardiovascular disease risk [55]. However, the tocopherols exert different anti-inflammatory [56, 57] and antioxidative properties [54], potentially necessitating a reconsideration of their respective impact on health and disease. One of the interesting findings in our study is that in response to the NCT, higher plasma γ-tocopherol concentrations correlate with ameliorated inflammatory response as reflected by reduced responses of pro-inflammatory chemokines and family members and ligands of the tumor necrosis factor receptor superfamily (TNFRSF) (BMP7, INHBA, COL8A1, and GFRA1). This may be of interest especially in view of the study population being overweight and obese men with low-grade inflammation who may have an elevated risk for development of type 2 diabetes (T2DM) and complications thereof such as cardiovascular disease and microvascular complications in kidney, eye, and extremities. Vitamin status and vitamin supplement interventions have received considerable attention in the field of T2DM [58, 59]. Low status of the antioxidants (vitamin A, C, and E) as well as low status of the B vitamins has been described in T2DM patients and is linked to increased risk of developing T2DM. Furthermore, clinical studies with vitamin E supplements in the form of α-tocopherol have resulted in varying outcomes.

Interestingly, we observed a specific overlap in regulation of thyroid-stimulating hormone beta (TSHB) by vitamin A, vitamin D_3_, and γ-tocopherol. For vitamin A (in rats) and D_3_ (in human), it has been shown that there is an association between low serum levels and high thyrotropin (TSH) concentrations [60, 61]. This is consistent with the possibility that vitamin A deficiency suppresses activation of pituitary retinoid receptor, thereby increasing TSHB mRNA transcription which leads to high TSH secretion [62]. For γ-tocopherol, there is no direct evidence of a relationship with thyroid-stimulating hormone (TSH). Thyroid hormones play an important role in regulating energy metabolism and thereby bodyweight and adipose tissue homeostasis and eventually insulin resistance. Therefore, their serum levels have been associated with cardiovascular risk factors in overweight and obese adolescents [63].

## Conclusions

Although this study is an observational study, it has resulted in a number of insights in the biological roles and importance of the eight micronutrients studied in homeostasis and more importantly in health with regard to phenotypic flexibility. Many of the correlations and expected observations could be confirmed in our study, providing confidence in the systems biology approach that was used. Interestingly, new insights of potential roles of micronutrients in health were found especially in response to a nutritional challenge test. Many of these correlations may only become clear when challenging the biological system as we did using the NCT. In the current study, we focused on the interpretation of the role of the isolated micronutrients. A next step would be to also consider multi-micronutrient interaction networks and its impact on health in which the current study limitations are taken into consideration such as genetics, where many single nucleotide polymorphisms can be linked to carotenoid metabolism. For example, lycopene bioavailability is associated with 28 single nucleotide polymorphisms (SNPs) in 16 genes [64] and also food intake analysis (markers) for a better understanding of high inter-individual variability in responses. Future epidemiological and intervention research building upon these insights by applying systems biology concepts, more extensive multi-nutrient analyses, and nutritional challenge tests will help to produce more efficient and potentially personalized micronutrient supplementation programs.

## Additional files

Additional file 1: Table S1. Nutritional composition of nutritional challenge test (NCT). (DOC 18 kb)
Additional file 2: Table S2. Plasma vitamin and carotenoid levels coherence. (DOC 18 kb)
Additional file 3: Figure S1. Box plot graphs representing the distribution of the values of selected parameters for tertiles of low, medium, and high micronutrient concentrations. (DOC 440 kb)

## References

[1] Kraemer K, Semba RD, Eggersdorfer M, Schaumberg DA (2012). Introduction: the diverse and essential biological functions of vitamins. Ann Nutr Metab.

[2] van Ommen B, Fairweather-Tait S, Freidig A, Kardinaal A, Scalbert A, Wopereis S (2008). A network biology model of micronutrient related health. Br J Nutr.

[3] Gammone MA, Riccioni G, D’Orazio N (2015). Carotenoids: potential allies of cardiovascular health?. Food Nutr Res.

[4] West KP, Pokhrel RP, Katz J, LeClerq SC, Khatry SK, Shrestha SR (1991). Efficacy of vitamin A in reducing preschool child mortality in Nepal. Lancet.

[5] Christian P, Khatry SK, Katz J, Pradhan EK, LeClerq SC, Shrestha SR (2003). Effects of alternative maternal micronutrient supplements on low birth weight in rural Nepal: double blind randomised community trial. BMJ.

[6] United Nations Standing Committee on Nutrition (2012). United Nations Standing Committee on Nutrition: what progress in nutrition?. Nutr Rev.

[7] Cole RN, Ruczinski I, Schulze K, Christian P, Herbrich S, Wu L (2013). The plasma proteome identifies expected and novel proteins correlated with micronutrient status in undernourished Nepalese children. J Nutr.

[8] WHO (2006). Constitution of the world health organization.

[9] Jadad AR, O’Grady L (2008). How should health be defined?. BMJ.

[10] Huber M, Knottnerus JA, Green L, van der Horst H, Jadad AR, Kromhout D (2011). How should we define health?. BMJ.

[11] van Ommen B, Keijer J, Heil SG, Kaput J (2009). Challenging homeostasis to define biomarkers for nutrition related health. Mol Nutr Food Res.

[12] van Ommen B, van der Greef J, Ordovas JM, Daniel H (2014). Phenotypic flexibility as key factor in the human nutrition and health relationship. Genes Nutr.

[13] Wopereis S, Rubingh CM, van Erk MJ, Verheij ER, van Vliet T, Cnubben NHP (2009). Metabolic profiling of the response to an oral glucose tolerance test detects subtle metabolic changes. PLoS One.

[14] Pellis L, van Erk MJ, van Ommen B, Bakker GCM, Hendriks HFJ, Cnubben NHP (2012). Plasma metabolomics and proteomics profiling after a postprandial challenge reveal subtle diet effects on human metabolic status. Metabolomics.

[15] Kardinaal AFM, van Erk MJ, Dutman AE, Stroeve JHM, van de Steeg E, Bijlsma S (2015). Quantifying phenotypic flexibility as the response to a high-fat challenge test in different states of metabolic health. FASEB J.

[16] Stroeve JHM, van Wietmarschen H, Kremer BHA, van Ommen B, Wopereis S (2015). Phenotypic flexibility as a measure of health: the optimal nutritional stress response test. Genes Nutr.

[17] Morine MJ, Monteiro JP, Wise C, Teitel C, Pence L, Williams A (2014). Genetic associations with micronutrient levels identified in immune and gastrointestinal networks. Genes Nutr.

[18] Kaput J, van Ommen B, Kremer B, Priami C, Monteiro JP, Morine M (2014). Consensus statement understanding health and malnutrition through a systems approach: the ENOUGH program for early life. Genes Nutr.

[19] Kaddurah-Daouk R, Kristal BS, Weinshilboum RM (2008). Metabolomics: a global biochemical approach to drug response and disease. Annu Rev Pharmacol Toxicol.

[20] Naylor S, Culbertson AW, Valentine SJ (2008). Towards a systems level analysis of health and nutrition. Curr Opin Biotechnol.

[21] Barnes S, Kim H (2004). Nutriproteomics: identifying the molecular targets of nutritive and non-nutritive components of the diet. J Biochem Mol Biol.

[22] Schweigert FJ (2007). Nutritional proteomics: methods and concepts for research in nutritional science. Ann Nutr Metab.

[23] Zhang X, Yap Y, Wei D, Chen G, Chen F (2008). Novel omics technologies in nutrition research. Biotechnol Adv.

[24] Bakker GC, van Erk MJ, Pellis L, Wopereis S, Rubingh CM, Cnubben NH (2010). An antiinflammatory dietary mix modulates inflammation and oxidative and metabolic stress in overweight men: a nutrigenomics approach. Am J Clin Nutr.

[25] Gold L, Ayers D, Bertino J, Bock C, Bock A, Brody EN (2010). Aptamer-based multiplexed proteomic technology for biomarker discovery. PLoS One.

[26] Kraemer S, Vaught JD, Bock C, Gold L, Katilius E, Keeney TR (2011). From SOMAmer-based biomarker discovery to diagnostic and clinical applications: a SOMAmer-based, streamlined multiplex proteomic assay. PLoS One.

[27] Vaught JD, Bock C, Carter J, Fitzwater T, Otis M, Schneider D (2010). Expanding the chemistry of DNA for in vitro selection. J Am Chem Soc.

[28] Lauridsen C, Halekoh U, Larsen T, Jensen SK (2010). Reproductive performance and bone status markers of gilts and lactating sows supplemented with two different forms of vitamin D. J Anim Sci.

[29] Aebischer CP, Schierle J, Schüep W (1999). Simultaneous determination of retinol, tocopherols, carotene, lycopene, and xanthophylls in plasma by means of reversed-phase high-performance liquid chromatography. Methods Enzymol.

[30] Shannon P, Markiel A, Ozier O, Baliga NS, Wang JT, Ramage D (2003). Cytoscape: a software environment for integrated models of biomolecular interaction networks. Genome Res.

[31] Olmedilla B, Granado F, Southon S, Wright AJ, Blanco I, Gil-Martinez E (2001). Serum concentrations of carotenoids and vitamins A, E, and C in control subjects from five European countries. Br J Nutr.

[32] Péter S, Moser U, Pilz S, Eggersdorfer M, Weber P (2013). The challenge of setting appropriate intake recommendations for vitamin E: considerations on status and functionality to define nutrient requirements. Int J Vitam Nutr Res.

[33] Hilger J, Friedel A, Herr R, Rausch T, Roos F, Wahl DA (2014). A systematic review of vitamin D status in populations worldwide. Br J Nutr.

[34] Jiang Q, Christen S, Shigenaga MK, Ames BN (2001). {gamma}-Tocopherol, the major form of vitamin E in the US diet, deserves more attention. Am J Clin Nutr.

[35] Institute of Medicine (US) (2000). Panel on Dietary Antioxidants, and Related Compounds. Dietary reference intakes for vitamin C, vitamin E, selenium, and carotenoids: a report of the Panel on Dietary Antioxidants and Related Compounds, Subcommittees on Upper Reference Levels of Nutrients.

[36] Lips P (2001). Vitamin D deficiency and secondary hyperparathyroidism in the elderly: consequences for bone loss and fractures and therapeutic implications. Endocr Rev.

[37] Liebler DC, Kaysen KL, Kennedy TA (1989). Redox cycles of vitamin E: hydrolysis and ascorbic acid dependent reduction of 8a-(alkyldioxy) tocopherones. Biochemistry.

[38] Wang X, Quinn PJ (1999). Vitamin E and its function in membranes. Prog Lipid Res.

[39] Mayilyan KR (2012). Complement genetics, deficiencies, and disease associations. Protein Cell.

[40] Espe K, Galler A, Raila J, Kiess W, Schweigert FJ (2007). High-normal C-reactive protein levels do not affect the vitamin A transport complex in serum of children and adolescents with type 1 diabetes. Pediatr Res.

[41] Abraham K, Müller C, Grüters A, Wahn U, Schweigert FJ (2003). Minimal inflammation, acute phase response and avoidance of misclassification of vitamin A and iron status in infants--importance of a high-sensitivity C-reactive protein (CRP) assay. Int J Vitam Nutr Res.

[42] Brown CC, Noelle RJ (2015). Seeing through the dark: new insights into the immune regulatory functions of vitamin A. Eur J Immunol.

[43] Chen J, He J, Hamm L, Batuman V, Whelton PK (2002). Serum antioxidant vitamins and blood pressure in the United States population. Hypertension.

[44] Rydén M, Garvin P, Kristenson M, Leanderson P, Ernerudh J, Jonasson L (2012). Provitamin A carotenoids are independently associated with matrix metalloproteinase-9 in plasma samples from a general population. J Intern Med.

[45] Hozawa A, Jacobs DR, Steffes MW, Gross MD, Steffen LM, Lee D-H (2009). Circulating carotenoid concentrations and incident hypertension: the Coronary Artery Risk Development in Young Adults (CARDIA) study. J Hypertens.

[46] Morrissey PA, Kiely M, Caballer B, Allen L, Prentice A (2005). Vitamin E: physiology and health effects. Encycl. Hum. Nutr.

[47] Mendivil CO, Zheng C, Furtado J, Lel J, Sacks FM (2010). Metabolism of very-low-density lipoprotein and low-density lipoprotein containing apolipoprotein C-III and not other small apolipoproteins. Arterioscler Thromb Vasc Biol.

[48] Borel P, Moussa M, Reboul E, Lyan B, Defoort C, Vincent-Baudry S, Maillot M, Gastaldi M, Darmon M, Portugal H, Lairon DPR (2009). Human fasting plasma concentrations of vitamin E and carotenoids, and their association with genetic variants in apo C-III, cholesteryl ester transfer protein, hepatic lipase, intestinal fatty acid binding protein and microsomal triacylglycerol transfer p. Br J Nutr.

[49] Olmedilla B, Granado F, Gil-Martinez E, Blanco I, Rojas-Hidalgo E (1997). Reference values for retinol, tocopherol, and main carotenoids in serum of control and insulin-dependent diabetic Spanish subjects. Clin Chem.

[50] Seo JY, Masamune A, Shimosegawa T, Kim H (2009). Protective effect of lycopene on oxidative stress-induced cell death of pancreatic acinar cells. Ann N Y Acad Sci.

[51] Gerhauser C (2013). Cancer chemoprevention and nutriepigenetics: state of the art and future challenges. Top Curr Chem.

[52] Han GM, Meza JL, Soliman GA, Islam KM, Watanabe-Galloway S (2016). Higher levels of serum lycopene are associated with reduced mortality in individuals with metabolic syndrome. Nutr Res.

[53] EFSA Panel on Dietetic Products, Nutrition, and Allergies (NDA). Scientific opinion on dietary reference values for vitamin E as α-tocopherol. EFSA J. 2015;13:4149.

[54] Hensley K, Benaksas EJ, Bolli R, Comp P, Grammas P, Hamdheydari L (2004). New perspectives on vitamin E: gamma-tocopherol and carboxyelthylhydroxychroman metabolites in biology and medicine. Free Radic Biol Med.

[55] Wagner K-H, Kamal-Eldin A, Elmadfa I (2004). Gamma-tocopherol—an underestimated vitamin?. Ann Nutr Metab.

[56] Reiter E, Jiang Q, Christen S (2007). Anti-inflammatory properties of alpha- and gamma-tocopherol. Mol Aspects Med.

[57] Cook-Mills JM. Isoforms of Vitamin E Differentially Regulate PKC α and Inflammation: A Review. J Clin Cell Immunol. 2013, 14;4(137). See also PMID: 23977443. 10.4172/2155-9899.1000137 PMC3748943 23977443

[58] Kaur B, Henry J (2014). Micronutrient status in type 2 diabetes: a review. Adv Food Nutr Res.

[59] Valdés-Ramos R, Guadarrama-López AL, Martínez-Carrillo BE, Benítez-Arciniega AD (2015). Vitamins and type 2 diabetes mellitus. Endocr Metab Immune Disord Drug Targets.

[60] Oba K, Kimura S (1980). Effects of vitamin A deficiency on thyroid function and serum thyroxine levels in the rat. J Nutr Sci Vitaminol.

[61] Mackawy AMH, Al-Ayed BM, Al-Rashidi BM (2013). Vitamin d deficiency and its association with thyroid disease. Int J Health Sci.

[62] Zimmermann MB, Wegmüller R, Zeder C, Chaouki N, Torresani T (2004). The effects of vitamin A deficiency and vitamin A supplementation on thyroid function in goitrous children. J Clin Endocrinol Metab.

[63] Souza L, Guedes E, Teixeira P, Moreira R, Godoy-Matos A, Vaisman M. Serum TSH levels are associated with cardiovascular risk factors in overweight and obese adolescents. J Pediatr (Rio J). 2016;92(5):532-8. 10.1016/j.jped.2016.01.011 27343633

[64] Borel P, Desmarchelier C, Nowicki M, Bott R (2015). Lycopene bioavailability is associated with a combination of genetic variants. Free Radic Biol Med.

