# Supplementary material for: The impact of micronutrient status on health: correlation network analysis to understand the role of micronutrients in metabolic-inflammatory processes regulating homeostasis and phenotypic flexibility
Source: Genes Nutr. 2017 Feb 8;12:5. doi: 10.1186/s12263-017-0553-7 (PMC5299688; doi:10.1186/s12263-017-0553-7)
Supplement: Additional file 1: Table S1. — Nutritional composition of nutritional challenge test (NCT). (DOC 18 kb) [file 12263_2017_553_MOESM1_ESM.docx]

**Table S1.**Plasma vitamin levels coherence

| **Nutritional value** |  | **Content** | **Energy** |
| --- | --- | --- | --- |
| Total |  |  | 2945kJ or 706kcal |
| Total protein |  | 20.7g | 11.7E% |
| Total fat |  | 46.1g | 58.7E% |
|  | Saturated fatty acids | 27.1g |  |
|  | Monounsaturated fatty acids | 11.8g |  |
|  | Polyunsaturated fatty acids | 1.4g |  |
|  | Cholesterol | 0.1g |  |
| Total carbohydrates |  | 52.2g | 29.6E% |
| Total mono-disaccharides |  | 42.3g |  |
| Total fiber |  | 0.1g |  |

*g* gram, *kj* kilojoule, *kcal* kilocalories, *E%* energy percent

From [15]**;** Pellis L et al. Metabolomics. 2012;8:347–59.
