# Supplementary material for: The impact of micronutrient status on health: correlation network analysis to understand the role of micronutrients in metabolic-inflammatory processes regulating homeostasis and phenotypic flexibility
Source: Genes Nutr. 2017 Feb 8;12:5. doi: 10.1186/s12263-017-0553-7 (PMC5299688; doi:10.1186/s12263-017-0553-7)
Supplement: Additional file 2: Table S2. — Plasma vitamin and carotenoid levels coherence. (DOC 18 kb) [file 12263_2017_553_MOESM2_ESM.docx]

**Table S2**: Plasma vitamin and carotenoid levels coherence. Spearman correlation analysis on plasma levels for all subjects included.

| Variable | Retinol | γ-Tocopherol | α-Tocopherol | β-Cryptoxanthin | Lycopene | α-Carotene | β-Carotene | 25-hydroxy-vitamin D3 |
| --- | --- | --- | --- | --- | --- | --- | --- | --- |
| Retinol | - | 0,303 | 0,720^***^ | 0,224 | 0,234 | 0,118 | 0,227 | 0,208 |
| γ-Tocopherol |  | - | 0,510^**^ | 0,181 | 0,303 | 0,155 | 0,024 | 0,208 |
| α-Tocopherol |  |  | - | 0,226 | 0,283 | 0,235 | 0,318 | 0,144 |
| β-Cryptoxanthin |  |  |  | - | 0,230 | 0,240 | 0,026 | -0,373^*^ |
| Lycopene |  |  |  |  | - | 0,284 | 0,334 | -0,242 |
| α-Carotene |  |  |  |  |  | - | 0,418^*^ | 0,215 |
| β-Carotene |  |  |  |  |  |  | - | 0,262 |
| 25-hydroxy vitamin D3 |  |  |  |  |  |  |  | - |

^*^p<0.05
^**^p<0.01
^***^p<0.001
