# Supplementary material for: The impact of micronutrient status on health: correlation network analysis to understand the role of micronutrients in metabolic-inflammatory processes regulating homeostasis and phenotypic flexibility
Source: Genes Nutr. 2017 Feb 8;12:5. doi: 10.1186/s12263-017-0553-7 (PMC5299688; doi:10.1186/s12263-017-0553-7)
Supplement: Additional file 3: Figure S1. — Box plot graphs representing the distribution of the values of selected parameters for tertiles of low, medium, and high micronutrient concentrations. (DOC 440 kb) [file 12263_2017_553_MOESM3_ESM.docx]

**Figure S1.** Box plot graphs representing the distribution of the values of selected parameters for tertiles of low, medium and high micronutrient concentrations. The graphs show: complement subunit-1r (C1r) **(A)** and high sensitive C-reactive protein (hsCRP) **(B)** correlating with retinol; total cholesterol **(C)**, and apolipoproteinC-III (ApoCIII) **(D)** correlating with α-tocopherol; insulin **(E),** and triglyceride C54:4 (TG-C54:4) **(F)**, correlating with γ-tocopherol; systolic blood pressure **(G),** and Plasminogen Activator Inhibitor-1 (PAI-1) **(H)** correlating with β-carotene; Interleukin-34 (IL-34) **(I)** and keratin-18 **(J)** correlating with α-carotene; Prostaglandin H2 synthase 2 (COX2) **(K),** and topoisomerase **(L)** correlating with lycopene; CD40 molecule (CD40) **(M)** and alanine aminotransferase (ALT) **(N)** correlating with β-cryptoxanthine; kallikrein 11 **(O)** and Interleukin-5 (IL-5) **(P)** correlating with vitamin D3. The X-axis separates the low 25% vitamin concentrations (1), the medium 50% concentrations (2), and the highest 25% vitamin concentrations. The Y-axis represent plasma concentrations of hsCRP (μg/L), total cholesterol (mmol/L), insulin (mU/L), and ALT (μg/L), systolic blood pressure (mmHg), and arbitrary units (relative concentrations to an internal standard) for all other parameters. The boxes represent the distribution of values (p25-p75) measured and the median (line), with standard deviation.


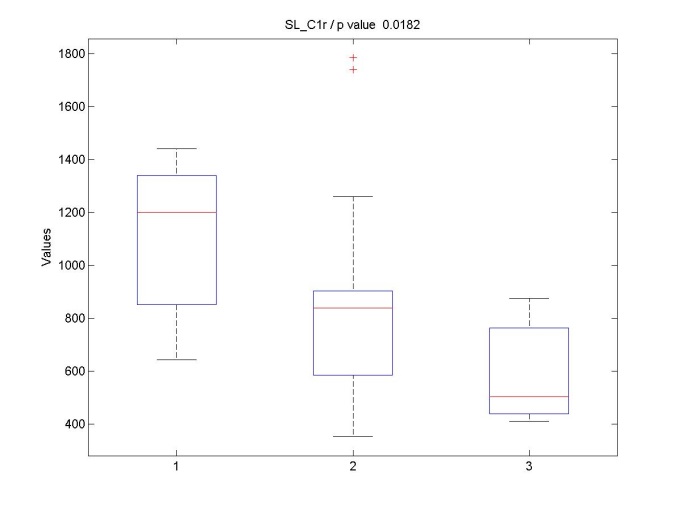

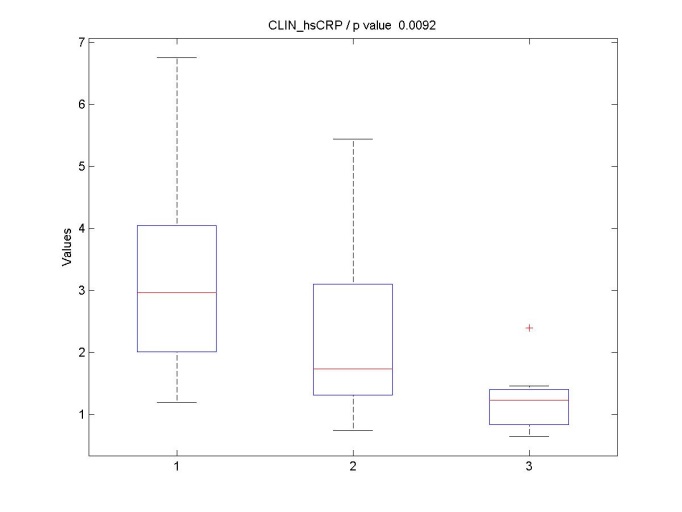

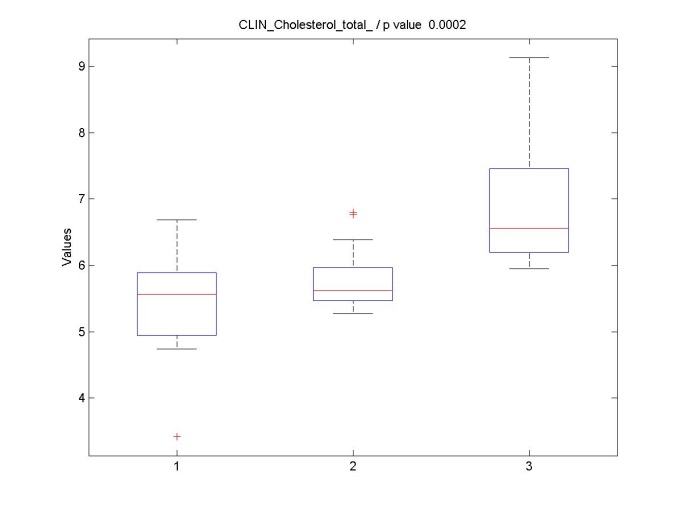

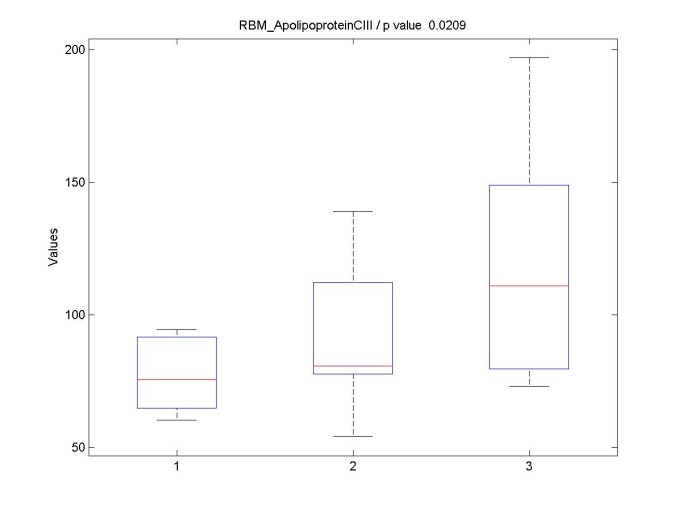

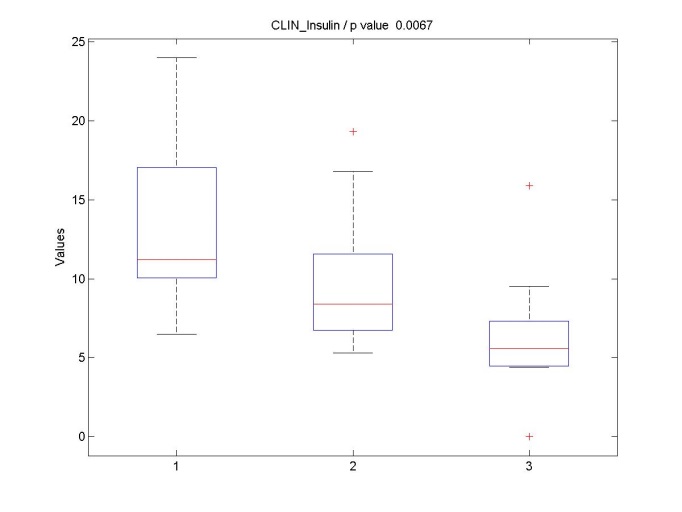

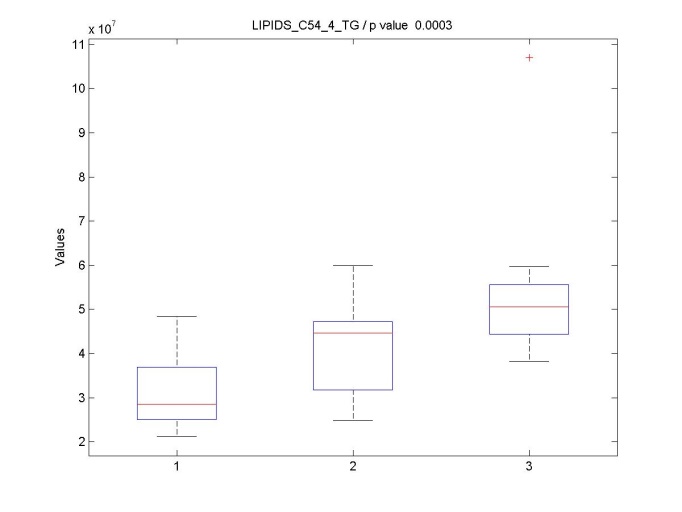


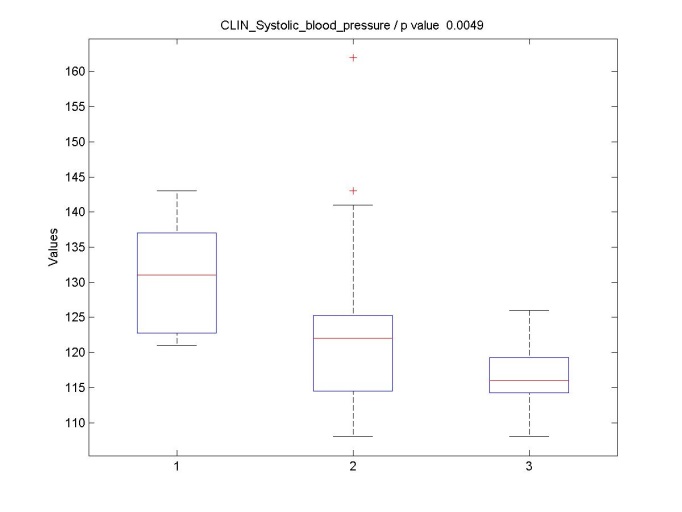

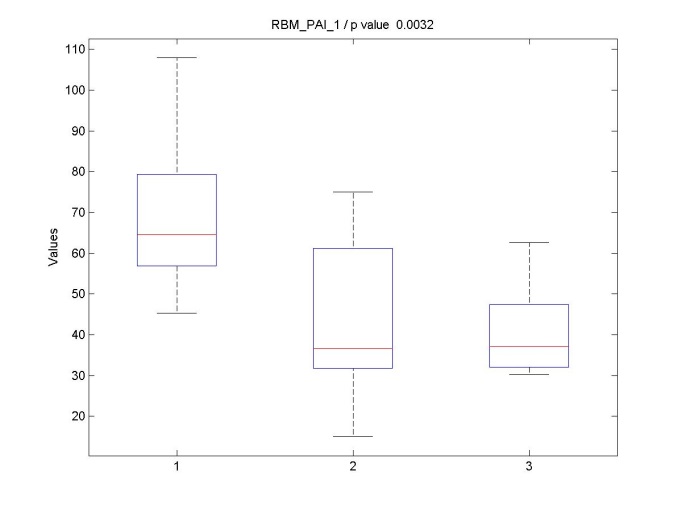

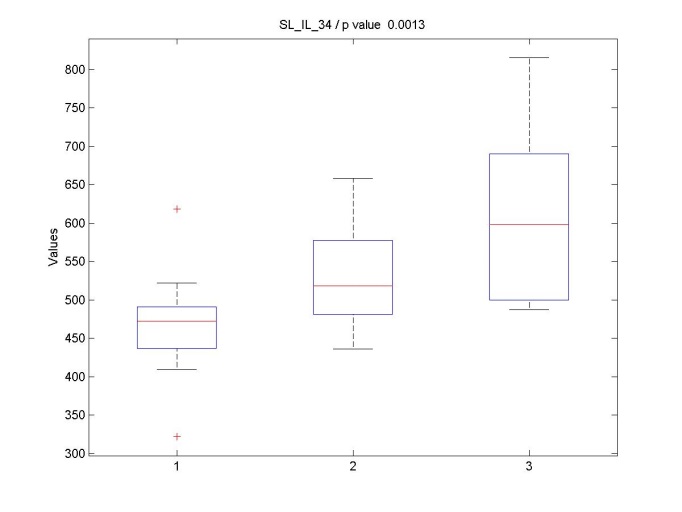

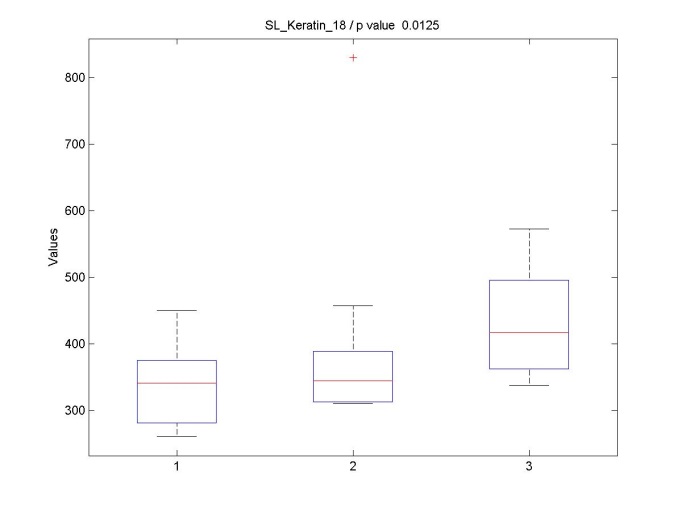

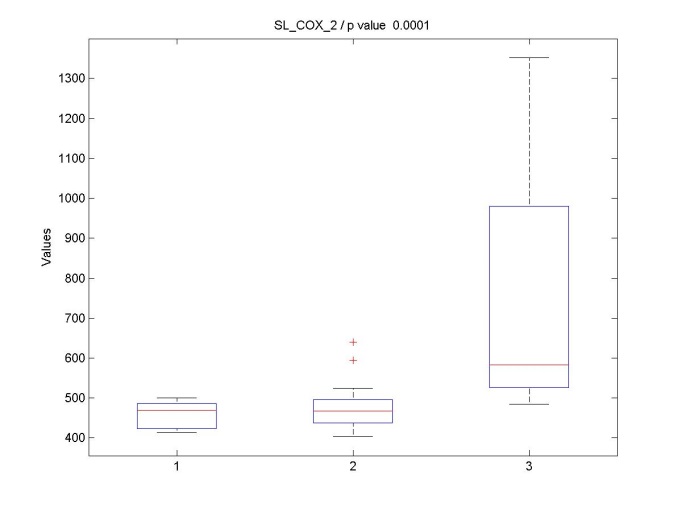

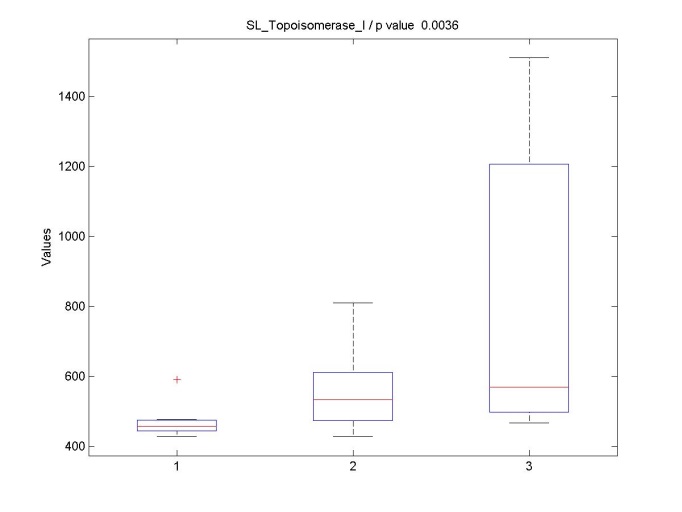

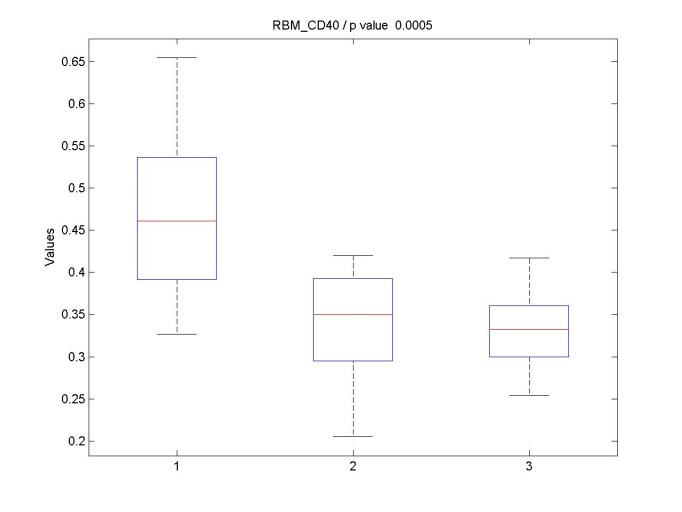

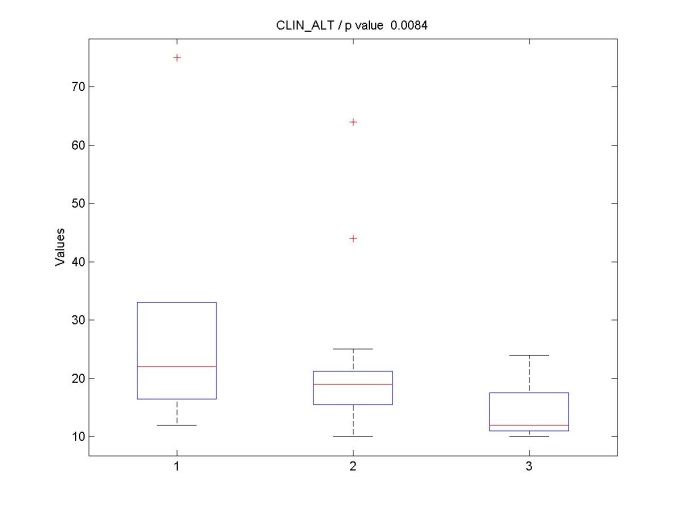


D

C

A

B

E

F

I

J

G

H

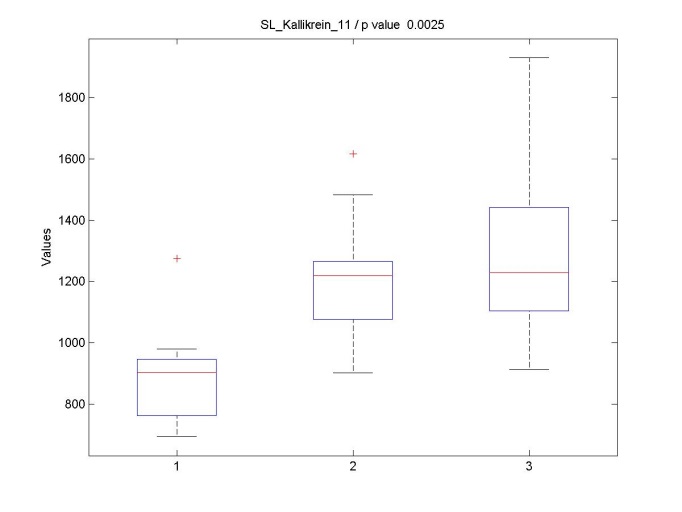

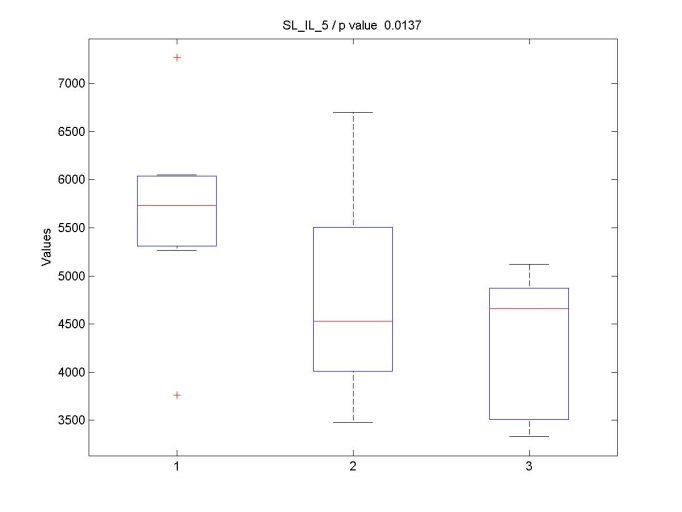


M

N

L

K

P

O
